# Supplementary material for: Efficacy and Safety of Sesame Oil Cake Extract on Memory Function Improvement: A 12-Week, Randomized, Double-Blind, Placebo-Controlled Pilot Study
Source: Nutrients. 2021 Jul 28;13(8):2606. doi: 10.3390/nu13082606 (PMC8399671; doi:10.3390/nu13082606)
Supplement: Supplementary file 1 [file nutrients-13-02606-s001.zip › nutrients-1292231-supplementary.pdf]

Supplementary materials

A. Amyloid β (1-40)

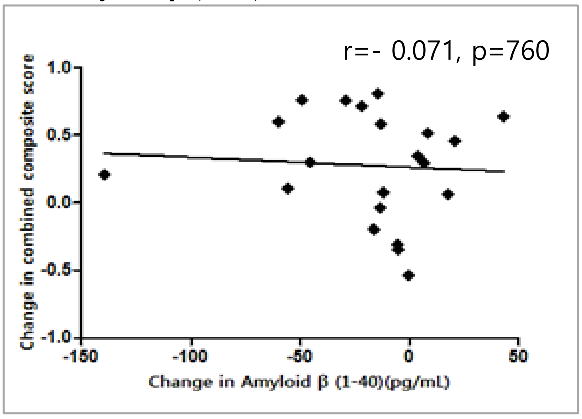

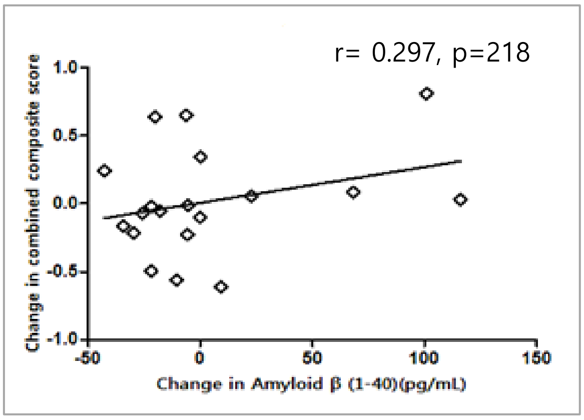

B. Amyloid β (1-42)

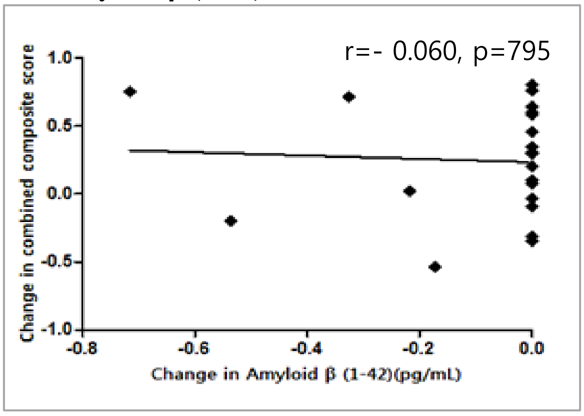

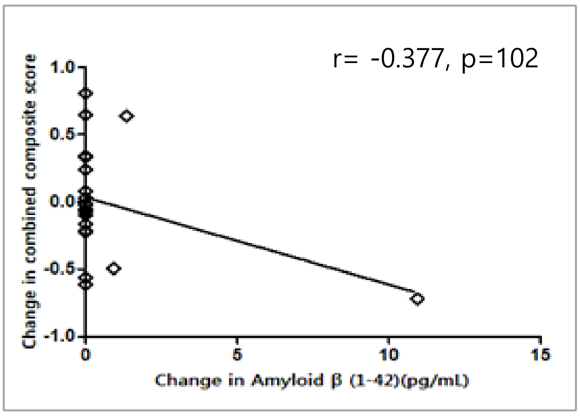

C. Amyloid β (42/40 ratio)

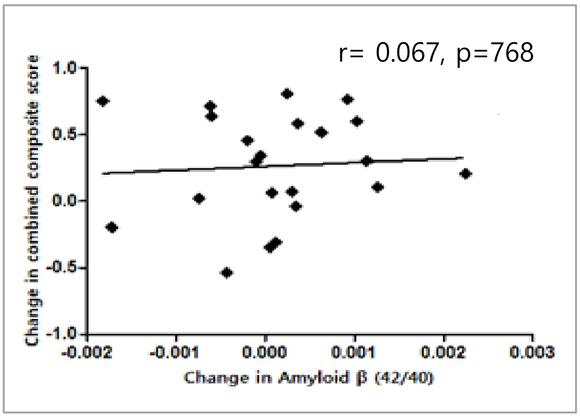

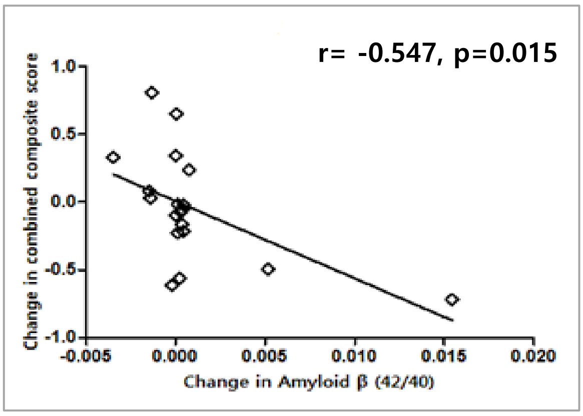

SOCE group **Placebo group**

Supplementary Figure S1. Associations between Changes in plasma amyloid-β levels and combined composite score

Supplementary Table S1. Major nutrient intakes and metabolic equivalent values of the **SOCE** and placebo groups measured at baseline and 12 weeks

	SOCE group (n=35)				Placebo group (n=35)				
	**Baseline**	**Week 12**	**Change**	***P* value[1]**	**Baseline**	**Week 12**	**Change**	***P* value[1]**	***P* value[2]**
Energy (Kcal)	1,534.2±546.5	1,471.3±499.8	62.9±391.5	0.348	1527.9±367.0	1,600.0±444.3	72.2±471.6	0.372	0.197
Carbohydrates (g)	251.9±91.3	244.5±73.9	-7.4±61.6	0.483	261.2±71.5	269.4±86.3	8.2±88.6	0.586	0.395
Lipids (g)	33.7±18.5	32.5±17.9	-1.2±20.2	0.732	31.6±14.8	34.0±12.3	2.4±17.2	0.426	0.435
Protein (g)	61.8±28.8	55.8±22.7	-6.0±26.9	0.196	55.8±17.1	59.7±18.3	3.9±19.4	0.239	0.081
Fiber (g)	22.8±10.4	21.5±9.9	-1.3±9.8	0.437	22.1±9.6	22.1±8.7	0.04±9.5	0.978	0.562
Vitamin A (ug RE)	804.9±592.2	872.9±603.2	68.0±624.8	0.524	914.2±682.5	901.5±618.2	-12.7±739.2	0.919	0.623
Vitamin D (ug)	3.8±5.3	4.6±6.4	0.9±8.6	0.554	3.9±5.3	4.6±5.9	0.7±7.6	0.602	0.925
Vitamin E (mg)	13.8±8.7	12.6±7.1	-1.2±8.8	0.429	13.3±6.0	13.7±7.6	0.3±7.8	0.800	0.446
Vitamin C (mg)	119.5±81.1	107.1±92.6	-12.4±74.7	0.333	110.5±100.7	110.9±68.1	0.4±97.4	0.982	0.540
MET value (min/week)	2,312.6± 2,916.1	2,670.9± 2,926.8	358.3± 1,840.6	0.258	2,605.7± 3,575.0	3,057.7± 2.915.8	452.0± 4.123.7	0.521	0.903

Values are presented as mean ± SD

[1] Analyzed by paired *t* test

[2] Change values between groups were analyzed by independent t-test

Abbreviation: MET, metabolic equivalent

Supplementary Table S2. Laboratory profiles of the subjects in this study

Laboratory profiles (standard range)	SOCE group (n=35)			Placebo group (n=35)			
	**Baseline**	**Week 12**	**p-value [1]**	**Baseline**	**Week 12**	**p-value [1]**	**p value[2]**
WBC (4.8–10.8×10³/μL)	5.8±1.2	5.6±1.4	0.360	5.8±1.8	5.5±1.1	0.215	0.681
RBC (4.2–5.4×100³/μL)	4.4±0.4	4.4±0.5	0.243	4.5±0.4	4.4±0.4	0.035	0.526
Hemoglobin (12–16g/dL)	13.6±1.2	13.4±1.3	0.145	13.8±1.3	13.6±1.3	0.014	0.520
Hematocrit (37–47%)	40.6±3.3	40.1±3.7	0.118	41.0±3.6	40.1±3.5	0.006	0.399
Platelet (130–450×10³/μL)	233.6±60.6	233.1±66.7	0.879	220.5±40.5	226.0±39.6	0.280	0.327
ALP (45–129IU/L)	71.3±23.5	69.5±24.6	0.252	65.6±15.9	64.2±16.6	0.200	0.787
GGT (8–48IU/L)	25.4±16.3	25.4±16.9	0.972	18.4±8.2	19.8±9.8	0.155	0.499
AST (12–33IU/L)	29.0±14.3	25.5±7.9	0.040	25.4±6.6	24.3±6.2	0.159	0.204
ALT (5–35IU/L)	26.5±11.7	22.9±10.7	0.031	24.2±12.6	22.6±12.0	0.267	0.353
Total bilirubin (0.2–1.2mg/dL)	0.8±0.3	0.8±0.2	0.216	0.9±0.3	0.8±0.3	0.039	0.265
Total protein (6.7–8.3g/dL)	7.4±0.4	7.2±0.4	0.000	7.4±0.5	7.3±0.4	0.053	0.178
Albumin (3.5–5.3g/dL)	4.4±0.2	4.4±0.3	0.014	4.4±0.2	4.3±0.2	0.047	0.600
BUN (8–23mg/dL)	15.8±4.2	16.5±4.2	0.347	17.3±4.2	17.6±4.1	0.534	0.705
Creatinine (0.7–1.7mg/dL)	0.7±0.2	0.7±0.2	0.019	0.8±0.2	0.8±0.2	0.880	0.156
Glucose (74–106mg/dL)	92.3±13.7	93.1±15.2	0.556	92.4±13.0	91.2±12.8	0.549	0.402
CK (50~200 IU/L)	112.9±110.9	94.8±37.5	0.312	119.3±77.1	98.2±50.2	0.039	0.882
LDH (218~472 IU/L)	365.8±46.8	372.5±51.9	0.880	375.0±62.5	359.3±51.6	0.010	0.012
pH (4.5~9.0)	6.2±0.8	6.0±0.7	0.162	6.4±0.8	6.3±0.7	0.328	0.889

Values are presented as mean ± SD

[1] Differences between baseline and 12 weeks within each group were analyzed by paired t-test

[2] Change values between groups were analyzed by independent t-test

Abbreviations: WBC,White Blood Cell; RBC, Red Blood Cell; ALP; Alkaline Phosphatase; GGT, Gamma Glutamyl Transferase; AST;Aspartate Transaminase; ALT,Alanine Transaminase; BUN, *Blood Urea Nitrogen; CK, creatine kinase; LDH, lactate dehydrogenase.*
